# Relationship between lipoprotein (a) and subclinical carotid atherosclerosis in asymptomatic individuals

**DOI:** 10.1016/j.clinsp.2022.100107

**Published:** 2022-09-27

**Authors:** Victor França, Érica Ivana Lázaro Gomes, Edite Vieira Silva de Campos, Vanessa Helena de Souza Zago, Valéria Sutti Nunes, Eliana Cotta de Faria

**Affiliations:** a Department of Clinical Pathology, Faculdade de Ciências Médicas da Universidade Estadual de Campinas, Campinas, SP, Brazil; b Pontifical Catholic University of Campinas, São Paulo, Brazil; c Laboratorio de Lipides (LIM10), Hospital das Clinicas HCFMUSP, Faculdade de Medicina, Universidade de Sao Paulo, Sao Paulo, SP, BR

**Keywords:** Lipoprotein (a), Carotid atherosclerosis, Carotid plaques

## Abstract

**Background:**

This study aimed to evaluate the associations between Lipoprotein (a) ‒ Lp(a) levels and carotid Intima-Media Thickness (cIMT) and with carotid plaques in healthy subjects because of previous contradictory data.

**Methods:**

A total of 317 healthy normolipidemic subjects (20‒77 years old) were selected. The cIMT and atherosclerotic plaques were determined by B-mode ultrasonography. Mann-Whitney tests were performed to compare the groups according to Lp(a) levels and to explore the associations between Lp(a), carotid plaques, and cIMT, logistic and linear regression analyses were performed.

**Results:**

Studied population (51% females, median age 43 years old) presented carotid plaques and cIMT ≥ 0.9 mm in 23% and 18% of the participants, respectively. The group with Lp(a) levels > 30 mg/dL presented significantly higher age and atherosclerotic plaques. Indeed, multivariate linear regression analysis showed a significant association between Lp(a), age, and race. On the other hand, logistic regression analysis demonstrated that the subjects with Lp(a) > 30 mg/dL have a significantly high risk of carotid plaques.

**Conclusion:**

The data from the present study indicate that Lp(a) levels above 30 mg/dL contribute to the development of carotid plaques even in apparently healthy participants.

## Highlights

• High lipoprotein (a) levels increase the risk of carotid plaques presence in asymptomatic subjects.
• Carotid intima-media thickness is not affected by plasma lipoprotein (a) levels.
• Age and race are directly associated with lipoprotein (a) levels, suggesting an important influence even in a highly miscegenated population.

## Introduction

Lipoprotein (a) ‒ Lp(a) is a lipoprotein fraction similar to the Low-Density Lipoprotein (LDL), that contains an apolipoprotein (a) linked to its structure. Plasma levels of Lp(a) display a wide range in the general population and is primarily controlled by hepatic synthesis of apo (a), which in turn varies according to the presence of polymorphisms in the *LPA* gene.^1^ Despite the similarities with plasminogen structure, apolipoprotein (a) contains only the Kringle (K) IV and V in its structure. Among the subtypes of KIV, specifically, subtype two of KIV (KIV2) presents a variable number of copies, reaching more than 40, thus it is possible to reach up to 34 different apo (a) isoforms.^2^^,^^3^

Levels of Lp(a) are considered an independent risk factor for cardiovascular disease according to epidemiologic and genetic studies carried out in different populations. In spite of it, reference risk values are not usually adopted.^1^^,^^4^^,^^5^

Lp(a) levels remain relatively constant in an individual throughout life and are not significantly affected by diet, age, and physical activity.^6^ Still, they vary according to ethnicity: individuals of African American descent have two to three times higher concentrations compared to Caucasians^7^ although Lp(a) levels may have a greater influence on plaque burden in white than in black individuals.^8^

The association of Lp(a) and cardiovascular diseases is justified by the atherogenic risk attached to the LDL structure and is aggravated by the similarity of apo (a) structure to plasminogen that could interfere in hemostasis. This effect may explain the role of Lp(a) in the promotion of cardiovascular diseases, eventually favoring atherogenic and prothrombotic processes.^1^^,^^9^

There is evidence that high Lp(a) levels, especially above 30 mg/dL, contribute to the progression of atherosclerotic plaque and increased carotid Intima-Media Thickness (cIMT).^10^, ^11^, ^12^, ^13^ However, this issue remains under debate. While some studies associate Lp(a) independently with cIMT and plaque development, other studies have not shown such an association.^14^, ^15^, ^16^, ^17^, ^18^ Furthermore, given the important role of Lp(a) in the development of cardiovascular diseases, the present study aimed to explore the association between Lp(a) levels and subclinical carotid atherosclerosis measured as cIMT as well as the presence of carotid plaques in asymptomatic individuals.

## Materials and methods

### Study population

This is a cross-section, retrospective study, for which were selected 317 individuals from both genders, aged between 19 and 77 years old, between the years 2008 and 2013, who participated in the FAPESP Thematic Project 2006/60585-9, after authorization by the Research Ethics Committee of the Faculty of Medical Sciences/UNICAMP. The study was carried out in accordance with the Declaration of Helsinki.

This project was carried out in partnership with the Municipal Health Department of Campinas, which provided the laboratory results of the lipid profile of individuals who spontaneously sought healthcare in the Basic Health Units of the city of Campinas, São Paulo, Brazil. As previously reported by Parra et al.^19^ the volunteers were initially selected according to plasma LDL-C (≤ 130 mg/dL) and triglycerides (≤ 150 mg/dL) levels. Thus, they were contacted by telephone and answered a screening questionnaire regarding medication use, Body Mass Index (BMI), and current and pre-existing diseases. The individuals who met the inclusion criteria were invited to attend clinical and laboratory evaluations.

On admission, all volunteers signed the Informed Consent Form (ICF) before a complete clinical, anthropometric, and laboratory evaluation. Peripheral venous blood samples were drawn in EDTA-containing tubes after a 12-hour fasting period. Serum and plasma were separated by centrifugation (4°C, 1000g, 10 minutes) and stored at -80°C until further analysis.

The inclusion criteria were both genders, BMI between 20 and 30 kg/m^2^; LDL-C ≤ 130 mg/dL and triglycerides ≤ 150 mg/dL levels, Systolic Blood Pressure (SBP) ≤ 140 mmHg or Diastolic Blood Pressure (DBP) ≤ 90 mmHg. Exclusion criteria comprised BMI higher than 30 kg/m^2^, regular use of medical treatment, especially those which interfere with lipids metabolism (statins, fibrates, oral contraceptives, and hormonal replacement therapy), and clinical and/or laboratory evidence of thyroid dysfunction, dyslipidemia, diabetes, metabolic syndrome, pregnancy, hypertension, liver, kidney or pulmonary disease, alcoholism, and smoking. Individuals who attend the inclusion criteria were invited to undergo carotid ultrasonography in a second visit.

### Clinical and anthropometrical evaluation

Volunteers answered a detailed questionnaire regarding their medical history, and their race was self-reported as white, black, or mixed.

Determinations included systolic and diastolic blood pressure (SBP and DBP, respectively) body weight (kg), height (cm), and circumferences of the waist and hips (WC, cm). Thus, BMI, waist-to-hip and waist-to-height ratios, all considered adiposity markers were calculated.^20^ The Lipid Accumulation Product (LAP) was also calculated according to the waist circumference and triglycerides levels (mmoL/dL) as described previously.^21^

### Biochemical analysis

Total Cholesterol (TC), High-Density Lipoprotein Cholesterol (HDL-C), triglycerides, glucose, uric acid, urea, alanine aminotransferase, aspartate aminotransferase, gamma-glutamyl transferase, creatinine and alkaline phosphatase were determined in the automatized system Modular® Analytics Evo (Roche Diagnostics, Burgess Hill, West Sussex, UK) using commercially available Roche Diagnostics® (Mannheim, Germany) assays. Thyroid Stimulation Hormone (TSH) and Thyroxin (T4L) were also determined in the automated system Elecsys 2010 (Roche, Basel, Switzerland) using Roche Diagnostics® (Mannheim, Germany) assays.

Low-Density Lipoprotein Cholesterol (LDL-C), and Very-Low Density Lipoprotein Cholesterol (VLDL-C) were determined by Friedewald's equation, and by triglycerides/5, respectively,^22^ Non-HDL Cholesterol (NHDL-C) were calculated by subtracting the HDL-C levels from total cholesterol.

Apolipoproteins A-I (apo A-I), B-100 (apo B-100) and [Lp(a)] were determined by nephelometry in the automated system BN II (Siemens Healthcare Diagnostics, Marburg, Germany) using commercially available assays (Dade-Boehringer®, Deerfield, IL, USA). C-Reactive Protein (CRP) was measured by immunoturbidimetry using the Tina-quant® CRP (latex) high-sensitivity assay (Roche Diagnostics®, Mannheim, Germany).

Plasma Paraoxonase-1 activity (PON1) was evaluated through an enzymatic method using paraoxon (diethyl-p-nitrophenylphosphate, Sigma, St. Louis, MO) as substrate.^23^

### Carotid Intima-Media Thickness (cIMT) determination and carotid plaques detection

The determinations of cIMT and plaques were performed by a single trained radiologist, blind to the study participants. Determinations were made by high-resolution B-mode ultrasonography using a 6–9 MHz linear array ultrasound imaging system ATL HDI 3500 Ultrasound System (Advanced Technology Laboratories Ultrasound, Bothell, USA). The volunteers were examined in dorsal decubitus and five longitudinal measurements of segments of left, and right common carotid arteries at the distal wall and 1 cm from the bifurcation were performed. Mean cIMT (mm) was calculated as the average of these measurements, and cIMT below 0.90 mm was considered a normal reference.^24^ In addition, it was considered as plaque the presence of at least one of the following parameters: focal wall thickening with at least 0.5 mm toward the vessel lumen, focal wall thickening at least 50% greater than that of the surrounding vessel wall, or focal region with cIMT above 1.5 mm that protrudes into the lumen that is distinct from the adjacent boundary.^25^^,^^26^

### Statistical analysis

The statistical analyses were performed using the software SAS 9.2 (SAS Institute Inc., Cary, NC, USA) and the SPSS 16.0 (SPSS Inc. Released 2007. SPSS for Windows, Version 16.0. Chicago, SPSS Inc.). A two-sided p-value ≤ 0.05 was considered statistically significant, and p-values > 0.05 and < 0.08 were determined as borderline.

Data normality was verified by the Shapiro-Wilk test. Due to the absence of normal distribution, numeric data were expressed as median and interquartile ranges and were adopted non-parametric tests. In order to explore the relationship between Lp(a) and the studied variables, the Spearman correlation test was performed. In addition, the parameters were compared by the Mann-Whitney test according to Lp(a) levels (≤ 30 mg/dL and > 30 mg/dL).

Univariate and multivariate linear regression analyses were performed to evaluate the influence of Lp(a) levels on the other studied parameters. Numeric variables were transformed into ranks due to the absence of normal distribution, and the results were as coefficients of determination (partial R^2^ and R^2^). Logistic regression analysis was performed in order to determine the Odds Ratio (OR) for plaque presence in the studied population.

## Results

Table 1 presents the descriptive analysis for clinical, anthropometrical, and biochemical parameters. The total population (n = 317; 51% female) was asymptomatic, with normal weight, blood pressure, and plasma lipids, in accordance with the respective guidelines adopted.^20^^,^^27^, ^28^, ^29^ Among the volunteers, 23% present increased cIMT (≥0.90 mm), and 18% were identified with carotid plaques.

**Table 1:** Anthropometric, clinical, and biochemical characteristics of individuals.

n	Median (25th‒75th)	Parameters
317	43 (34‒54)	Age (years)
163/154	‒	Sex (Female/Male)
259/58	‒	Race (White/Non-white)
317	23 (22‒25)	BMI (kg/m^2^)
317	74 (68‒81)	Waist circunference (cm)
316	92 (88‒98)	Hips circunference (cm)
316	0.8 (0.7‒0.9)	Waist-to-hip ratio
317	0.5 (0.4‒0.5)	Waist-to-height ratio
317	9 (4‒17)	LAP (cm/mmoL/L)
317	120 (110‒128)	SBP (mmHg)
317	80 (72‒80)	DBP (mmHg)
317	0.6 (0.5‒0.7)	Left cIMT (mm)
317	0.6 (0.5‒0.7)	Right cIMT (mm)
317	0.6 (0.5‒0.7)	Mean cIMT mm)
58/259	‒	Plaques (Yes/No)
317	173 (153‒198)	Total cholesterol (mg/dL)
317	58 (38‒74)	HDL-C (mg/dL)
317	117 (98‒135)	NHDL-C (mg/dL)
317	73 (55‒95)	Triglycerides (mg/dL)
317	101 (85‒120)	LDL-C (mg/dL)
317	15 (11‒19)	VLDL-C (mg/dL)
317	149 (118‒176)	ApoA-I (mg/dL)
317	78 (67‒93)	ApoB100 (mg/dL)
317	12 (4‒26)	Lp(a) (mg/dL)
313	0.8 (0.4‒1.7)	hsCRP (mg/L)
315	32 (13‒53)	PON1 (µmoL/min)
317	84 (78‒88)	Glucose (mg/dL)
317	30 (25‒35)	Urea (mg/dL)
317	0.8 (0.7‒0.9)	Creatinine (mg/dL)
317	4.5 (3.8‒5.4)	Uric acid (mg/dL)
317	17 (13‒22)	ALT (U/L)
297	19 (16‒23)	AST (U/L)
316	65 (52‒76)	ALP (U/L)
297	18 (13‒25)	GGT (U/L)
317	2 (1.4‒2.9)	TSH (uUI/mL)
317	1.3 (1.2‒1.4)	FT4 (ng/dL)

N, Sample size; Non-whites include: Blacks, mixed races, and asian; BMI, Body mass index; LAP, Lipid accumulation product; SBP, Systolic blood pressure; DBP, Dastolic blood pressure; cIMT, Carotid intima media thickness; HDL-C, High-density lipoprotein cholesterol; NHDL-C, Non-high density lipoprotein cholesterol; LDL-C, Low-density lipoprotein cholesterol; VLDL-C, Very low-density lipoprotein cholesterol; ApoA-I, Apolipoprotein A‒I; ApoB-100, Apolipoprotein B100; Lp(a), Lipoprotein (a); hsCRP, High sensitivity C-reactive protein; PON1, Paraoxonase-1; ALT, Alanine aminotransferase; AST, Aspartate aminotransferase; ALP, Alkaline phosphatase; GGT, Gamma glutamyl transferase; TSH, Thyroid stimulating hormone; FT4, Free thyroxine. Literature used for reference values: Brazilian Guidelines on Obesity 2016 4^th^ edition; World health organization ‒ average body mass index; Brazilian Guideline for Hypertension; Update of the Brazilian guideline on dyslipidemia and prevention of atherosclerosis 2017; Guideline of the Brazilian Diabetes Society; Clinical diagnosis and treatment by laboratory methods 21 edition; Clinical practice guideline for the assessment and management of chronic kidney disease; Brazilian consensus on thyroid. Values are expressed as median and interquartile range (25^th^ and 75^th^).

The variables were compared between groups stratified according to Lp(a) levels (Table 2). The group with levels above 30 mg/Dl (21%) exhibited a higher median age, apolipoprotein B-100, PON1 activity, and GGT levels. In addition, the presence of plaques and non-white individuals was also significantly more frequent in this group.

**Table 2:** Significant comparisons of demographic and biochemical variables of individuals by serum Lp(a) concentrations.

Parameters	Lp(a) ≤ 30 mg/dL)		Lp(a) > 30 mg/dL		p‒value
	n	Median (25^th^‒75^th^)	n	Median (25^th^‒75^th^)	
Age (years)	250	43 (33‒53)	67	45 (36‒57)	**0.04**
Race (White/No‒white)	215/35		44/23		**0.01**
Plaque (Yes/No)	40/210		18/49		**0.04**
Biochemical parameters					
ApoB100 (mg/dL)	250	76 (65‒91)	67	86 (75‒102)	**< 0.01**
hsCRP (mg/L)	247	0.80 (0.40‒1.50)	66	0.93 (0.50‒1.90)	0.07
PON1 (µmoL/min)	249	28 (12‒52)	66	42 (19‒57)	**0.01**
GGT (U/L)	236	17 (13‒23)	61	21 (15‒30)	**0.02**

N, Sample size; Lp(a), Lipoprotein (a); Non-whites included,: Blacks, mixed races, and asian; ApoB-100, Apolipoprotein B100; hsCRP, high sensitivity C-reactive protein; PON1, Activity paraoxonase 1; GGT, Gamma-glutamyltransferase; Values are expressed as interquartile ranges (25^th^ and 75^th^); significant p-value: ≤ 0.05; the numerical variables were compared by the Mann-Whitney test and the non‒numerical variables by Chi-Square; the variable Lp(a) was transformed into.

Spearman's correlation between Lp(a) and the other parameters revealed positive and significant correlations with age (R = 0.167; p < 0.01), SBP (R = 0.136; p < 0.02), DBP (R = 0.109; p = 0.05), ApoB-100 (R = 0.258; p < 0.01), PON1 (R = 0.108; p = 0.05), GGT (R = 0.123; p = 0.03), hsCRP (R = 0.157; p < 0.01), and a tendency towards cIMT (R = 0.099; p = 0.08).

The possible associations between Lp(a) and the other variables were explored in the analysis of univariate and multivariate linear regression, whose significant parameters are shown in Table 3. Direct and significant associations with age and race were observed, both in the univariate and multivariate analyses. SBP and DBP are also directly associated with Lp(a) in univariate analysis, however, they did not show significance in multivariate analysis. The LAP, an adiposity parameter, was indirectly related to Lp(a) in both univariate and multivariate analyses.

**Table 3:** Linear regression analysis between Lp(a) and clinical and anthropometric parameters.

	Univariate			Multivariate			
Parameters	n	Β	p-value	β	R^2^-partial	R^2^-total	p-value
Age (years)	317	1.21	**< 0.01**	1.35	**0.02**	**0.07**	**< 0.01**
Race (White/No-white)	317	34.73	**< 0.01**	32.07	**0.04**		**0.01**
LAP (cm/mmoL/L)	317	‒0.65	0.14	-0.87	**0.01**		**0.05**
SBP (mmHg)	317	0.93	**0.01**				
DBP (mmHg)	317	1.00	**0.02**				

Linear regression analysis; N, Number of individuals; β, Estimated parameter; significant p-value ≤ 0.05; Non-whites include blacks, mixed races and asian; LAP, Lipid accumulation product; SBP, Systolic blood pressure; DBP, Diastolic blood pressure.

Table 4 shows the analysis of univariate and multivariate logistic regression to explore the association between the presence of plaques, Lp(a), and other parameters. With each year of life, the odds ratio for plaque increases by about 1.11 times (11%). For non-white individuals, the odds ratio for the plaque is approximately 1.97 times (97%) in univariate, and 2.5 times (250%) in the multivariate analysis. Regarding Lp(a) concentrations above 30 mg/dL, there is a 2% increase in the odds ratio for plaque in univariate analysis.

**Table 4:** Univariate and multivariate logistic regression analysis for the presence of plaques.

	Univariate			Multivariate		
Parameters	OR	95% CI	p-value	OR	95% CI	p-value
Age (years)	1.11	1.08‒1.1	**< 0.01**	1.11	1.08‒1.1	**< 0.01**
Race (White/No-white)	1.97	1.0‒3.9	**0.05**	2.50	1.2‒5.6	**0.02**
Lp(a) (mg/dL)	1.02	1.0‒1.0	**0.02**			

OR, Odds Ratio; 95% CI, Confidence Interval 95%; Non-whites Include: blacks, mixed races and asian; Lp(a), Lipoprotein (a); p-value ≤ 0.05; Lp(a) was transformed into.

## Discussion

The present study investigated the possible relationships between plasma Lp(a) levels and subclinical atherosclerosis determined by cIMT, as well as the presence of carotid plaques in asymptomatic adults. The group selected for this study consisted of individuals of both genders, predominantly self-declared as white (82%) and clinically assessed as normotensive, normolipidemic, and eutrophic, with no evidence of pre-established cardiovascular diseases or other pathologies. In addition, the group consists of adults (with a median age of 43 years), of whom carotid plaques were reported in 18%.

The contribution of cIMT to the prediction of cardiovascular risk is widely reported in the literature, and the presence of plaques adds significant value to this determination illustrating the heterogeneity of the atherosclerotic disease. On the one hand, the cIMT reflects the thickening process of the intima-medial layer that normally increases with age and smooth muscle hypertrophy. In contrast, the presence of plaques is specific and shows the established atherosclerotic disease.^30^

The occurrence of carotid atherosclerotic plaques is recognized to increase with age, however, the prevalence in populations can vary significantly according to the methods applied for its determination and the definition of plaque adopted. Another sensitive point is the population itself, concerning the presence of comorbidities, lifestyle, and type of diet, among other factors. For example, Ihle-Hansen and colleagues^31^ evaluated cIMT and the presence of plaques in 3683 individuals aged between 63 and 65 years; in this group, 87% had carotid plaques. Not surprisingly, the presence of risk factors was significant, of which hypertension (62%), hypercholesterolemia (53%), obesity (23%), and diabetes mellitus (11%) stand out. In this sense, age plus the presence of these other well-established risk factors explain this high plaque prevalence. Other studies in the general population corroborate the high prevalence of atherosclerotic plaques according to the age group and the presence of other risk factors, whose severity increases significantly as these clinical aspects add up.^32^ Recently, Song P. and collaborators evaluated from a robust meta-analysis that the prevalence of atherosclerotic plaques in the population between 30 and 79 years is 21.13%, which is equivalent to more than 800 million people.^33^

Studies assessing the cIMT and the prevalence of plaques in asymptomatic individuals and their association with non-established risk factors such as Lp(a) are scarce. A study with 6617 Chinese, with no history of CVD, diabetes mellitus, hypertension, and smoking found a mean cIMT of 0.60 mm, considering their age was 49.7 ± 5.3.^34^ Regarding plaques, the prevalence was 9%, lower than that observed in the present study. This finding, on the one hand, points to the possibility of the presence of an underlying factor, hypothetically Lp(a), which, apart from traditional factors, may contribute to this relatively high prevalence. On the other hand, it shows the challenge in interpreting parameters that vary according to the technique used, location and number of measurements, number of individuals enrolled, and software algorithm analysis. In this sense, it is possible to state that cIMT and plaques are unlikely to be directly compared, even among groups of healthy individuals.

As expected, age was a factor strongly associated with carotid plaques in this study, although it was composed of healthy individuals. However, the association between Lp(a) levels higher than 30 mg/dL, and the odds ratio for plaques in the univariate analysis suggests a relationship that can be explored. Tsimikas and colleagues (1) reported the presence of apo (a) in atherosclerotic plaques, suggesting that Lp(a) in high concentrations may contribute to plaque progression. There was a positive association between Lp(a) and carotid atherosclerosis in adults with ischemic stroke aged 16 to 54 years in France.^12^ The Lp(a) has also been associated with the development of atherosclerosis in individuals with primary hypercholesterolemia and Lp(a) concentrations greater than 50 mg/dL in a group of individuals aged 18 to 80 years in Spain.^35^ Hippe and colleagues^13^ reported that Lp(a) concentrations greater than 64 mg/dL were independent predictors of carotid plaque progression in individuals with established cardiovascular disease, even maintaining serum LDL-C concentrations below 70 mg/dL. However, Huffman and colleagues^18^ did not find an association between Lp(a) concentrations and atherosclerosis among South Asians living in America aged 40 to 84 years without cardiovascular disease. Some studies independently associate Lp(a) with cIMT and carotid atherosclerotic plaques, other studies, however, do not show this correlation, and others in turn report that Lp(a) is more associated with established CVD than in asymptomatic individuals, once that Lp(a) concentrations tend to increase in chronic inflammatory processes, such as rheumatoid arthritis and systemic lupus erythematosus.^2^^,^^3^^,^^14^

In this study, no correlation was found between Lp(a) and cIMT. Nonetheless, Knoflach and collaborators^36^ found a direct association between Lp(a) and cIMT in asymptomatic Austrian women aged 18 to 22 similarly to the study by Schreiner and collaborators^37^ that found a positive correlation between Lp(a) and cIMT in Americans aged 45 to 64, suggesting that Lp(a) may be associated with development subclinical carotid atherosclerosis. However, Calmarza and colleagues^16^ did not find such an association in a study of 172 asymptomatic Spanish individuals aged 43 to 93 years. This association is, in fact, a sensitive point for analysis, since the increase in cIMT can be associated with carotid plaque, however, cIMT cannot be considered an independent predictor of the development of carotid plaque, although these events often coexist and share some vascular determinants common.^38^

It is agreed that the plasma concentrations of Lp(a) are genetically determined more specifically by the presence of polymorphisms in the *LPA* gene that determine the size of the type 2 kringle IV peptide chain (KIV-2) (3). Thus, the larger the size of KIV-2, the greater the Lp(a); in contrast, its plasma concentration is lower. This reverse process is associated with the easier removal of larger particles, while smaller ones tend to remain in circulation for longer periods.^9^ Evidence points to the participation of LDLR in the removal process, including as a therapeutic target for reducing plasma Lp(a) concentrations. Nonetheless, many contradictions regarding the results observed with statins and PCSK9 inhibitors must be considered.^1^^,^^3^

Given the complexity of this lipoprotein, reference values have not been defined for the general population. In fact, studies in different ethnic and population groups point to a wide range. Thus, some studies indicate that values above 50 mg/dL (125 nmoL/L) or the 80^th^ percentile are considered high.^9^ Other studies, on the other hand, adopt the 30 mg/dL cut.^1^^,^^8^^,^^9^^,^^39^, ^40^, ^41^, ^42^ In fact, 21% of the recruited individuals had Lp(a) above this value. Some studies show that around 30% of the world population has high Lp(a), which represents approximately two billion individuals.^1^^,^^9^^,^^18^ The Lp(a) concentrations in the asymptomatic individuals of the present study were expressed with a median of 12 mg/dL (4‒26^th^ percentiles), indicating great similarity with other studies.^43^ In a study with Brazilian individuals aged 46 to 68 years, to verify a possible association between concentrations of Lp(a) and coronary artery disease, the control group showed average concentrations of 11 mg/dL of Lp(a).^44^ Another study carried out in the city of Ouro Preto, state of Minas Gerais, Brazil, with 400 asymptomatic individuals, showed Lp(a) concentrations with a median of 18.11 mg/dL with an average age of 45 years.^45^ Schoolchildren averaging 10 years-old, also demonstrated Lp(a) concentrations with a median of 25.5 mg/dL in the Ouro Preto study.^45^ A recent study involving 4,140 individuals in a cardiovascular reference center in São Paulo, showed an average Lp(a) concentration of 24.8 mg/dL.^46^

In addition, interesting relationships were observed in this study between Lp(a) levels and race. Several shreds of evidence in the literature point to those non-white individuals having higher concentrations of Lp(a), about two to three times higher than Caucasians or Orientals, revealing that there is ethnic variability between different population groups, whose underlying causes have not yet been fully understood.^3^^,^^47^

It was possible to perceive in the present study that serum Lp(a) concentrations in non-white individuals were higher, with a median of 22.85 mg/dL (5‒36 percentiles), and in white individuals with a median of 11.10 mg/dL (4‒22 percentiles), with the significance level of p < 0.01 when compared by the Mann-Whitney test. These concentrations can be partially explained by variations in the *LPA* locus, mainly by the polymorphism of the apo (a) gene (LPA; MIM 152200). Among the variants, notably Schmidt and colleagues evaluated three SNPs and reported that they contribute to different concentrations of Lp(a) in African-American and Caucasian individuals.^47^ Two SNPs (T3888P and G + 1/inKIV-8A), suppressing the Lp(a) set, were more common in Caucasians, while the third SNP (G-21A) increased the activity of promoting apo (a), this being more common in African Americans.^3^

Other studies looked at different populations and found low concentrations also in non-Hispanic Caucasians with a median of 12 mg/dL (5‒32 percentiles), in Chinese with a median of 11 mg/dL (5‒26 percentiles), in Japanese with a median of 13 mg/dL (5‒26 percentiles), and in Hispanics with a median of 19 mg/dL (8‒43 percentiles).^1^^,^^3^^,^^9^^,^^47^ Even with different concentrations and isoforms in some populations, Lp(a) was associated positively and similarly with the risk of cardiovascular events in a 20-year longitudinal follow-up study among Caucasian individuals and individuals of African descent.^1^ Given this, there is still a lot of discussion about the concentrations considered adequate for Lp(a) due to the great miscegenation of the population, mainly in countries like Brazil.^6^

Other factors such as age, sex, and environmental conditions do not have a major influence on Lp(a) concentrations in most studies. However, in the present study, Lp(a) concentrations greater than 30 mg/dL were found in older individuals. Some studies suggest that among adults, Lp(a) concentrations have been somewhat variable, and an association between Lp(a) and longevity has been suggested among the elderly.^48^, ^49^, ^50^ On the other hand, a substantial number of studies report that there is not an association between age and Lp(a) concentrations, however, the question of the extent to which age can influence Lp(a) concentrations remains uncertain.^6^^,^^49^

In the present study, there was no significant difference between the concentrations of Lp(a) and gender. However, a meta-analysis with 36 studies showed that females had 12% higher concentrations of Lp(a) compared to males.^51^ Likewise, it occurred with the BMI, where there were no significant results for the BMI classes, but it was the LAP, also considered a parameter of adiposity, which was indirectly related to the Lp(a). In a Japanese study, with 2,997 asymptomatic individuals aged between 40 and 69 years, Lp(a) concentrations were shown lower in non-obese individuals (BMI ≥ 26).^52^

The concentration of GGT in the present study was directly correlated with the concentrations of Lp(a). Since there is a similarity between Lp(a) and the LDL particle, some studies propose that Lp(a) also undergoes an oxidative process as LDL, and during the atherosclerotic process and GGT activity can increase to compensate for the increase in oxidative stimulants, increasing the synthesis of glutathione, which is an important antioxidant.^1^^,^^53^ A study conducted by Ghatge and collaborators^54^ also found a positive correlation between the concentrations of Lp(a) and GGT.

In this study, PON1 also showed a significant correlation and increased activity in individuals whose Lp(a) concentrations were above 30 mg/dL. This was described in a study with individuals who had moderate to high concentrations of Lp(a).^55^ It is not yet clear whether PON1 concentrations may have any benefit in individuals with high concentrations of Lp(a).^55^ The serum concentrations of hsCRP were also correlated to Lp(a), and the comparison between individuals with the presence and absence of plaques was significant by the Man Whitney test (p < 0.02), which may suggest a possible underlying inflammatory process implied by atherosclerosis.^53^

The present study has certain limitations since it has not analyzed genetic factors that influence the concentration of Lp(a) and the identification of apo (a) isoforms. Another limitation is that only carotid ultrasound was performed and no other arterial systems susceptible to atherosclerosis were examined. Therefore, the outcome could have been different or increased from the present results if the coronary and femoral arteries had been analyzed. Nevertheless, a strict criterion was adopted for the selection of participants, which caused a limited number of individuals, and the analysis of Lp(a) was performed by nephelometry, considered the gold standard for analysis of Lp(a).

This study pointed out that Lp(a) concentrations above 30 mg/dL were associated with the presence of carotid plaques in asymptomatic individuals with a median age of 43 years, and a 2% odds ratio for the presence of plaques. In fact, the relationship between Lp(a) and CVD in general, is well established in the literature, however, the strength of these associations and the mechanism in which Lp(a) influences the atherosclerotic process must be explored further.

## Conflicts of interest

The authors declare no conflicts of interest.
